# Revisiting Kelvin equation and Peng–Robinson equation of state for accurate modeling of hydrocarbon phase behavior in nano capillaries

**DOI:** 10.1038/s41598-021-86075-8

**Published:** 2021-03-22

**Authors:** Ilyas Al-Kindi, Tayfun Babadagli

**Affiliations:** Department of Civil and Environmental Engineering, School of Mining and Petroleum Engineering, University of Alberta, 7-277 Donadeo Innovation Centre for Engineering, 9211-116th Street, Edmonton, AB T6G 1H9 Canada

**Keywords:** Energy science and technology, Engineering, Nanoscience and technology

## Abstract

The thermodynamics of fluids in confined (capillary) media is different from the bulk conditions due to the effects of the surface tension, wettability, and pore radius as described by the classical Kelvin equation. This study provides experimental data showing the deviation of propane vapour pressures in capillary media from the bulk conditions. Comparisons were also made with the vapour pressures calculated by the Peng–Robinson equation-of-state (PR-EOS). While the propane vapour pressures measured using synthetic capillary medium models (Hele–Shaw cells and microfluidic chips) were comparable with those measured at bulk conditions, the measured vapour pressures in the rock samples (sandstone, limestone, tight sandstone, and shale) were 15% (on average) less than those modelled by PR-EOS.

## Introduction

Steam injection is known as one of the traditional methods used to increase heavy-oil recovery by effectively lowering the oil viscosity via raising its temperature. The major drawback of steam injection is the massive energy required to heat the matrix, which usually acts as an energy sink^1^. Such a limitation causes steam-injection applications to be highly expensive projects. This issue has encouraged engineers and researchers to study other alternatives to enhance the mobility of heavy oil in porous matrixes. Using solvents in heavy-oil recovery has become a common thought, since injecting chemicals solely or as co-injectant with steam can improve oil recoveries. Different types of hydrocarbon and non-hydrocarbon solvents were considered previously due to their effective diffusion capabilities into crude oil and bitumen. The diffusion of solvents into the heavy oil results in a reduction of oil viscosity which makes it flow easier within the rock porous media.

Nasr et al.^2^ investigated the impact of co-injecting hydrocarbon solvents (propane to heptane range suggesting hexane as the optimal one) with steam in heavy oil and bitumen recovery during steam assisted gravity drainage (SAGD) operations. The research generally focused on improving oil rate, enhancing the oil steam ratio, reducing required energy, and dropping water consumption. The selection criteria of solvents was performed based on vaporization and condensation temperatures, and how close they were to the water vaporization and steam condensation temperatures. Léauté and Carey^3^ studied the impact of C5+ condensate on bitumen recovery in the cyclic steam stimulation (CSS) process for the Cold Lake field. The application was inspected in the field as a pilot project through eight wells under CSS operation. It was reported that adding 6% volume fraction of diluent into steam during CSS enhanced the well performance and results were above the researcher expectations.

Utilizing hydrocarbon solvents in heavy-oil recovery applications has a significant limitation due to operational cost. Injecting large volumes of solvents could be expensive and uneconomical in many circumstances, depending on oil prices. To minimize the overall application cost, Al-Bahlani and Babadagli^4^ introduced the idea of Steam-Over-Solvent in Fractured Reservoirs (SOS-FR) to retrieve the trapped solvents in the reservoirs thermally by either steam or hot-water injection. The process consists of three main stages: (1) injecting steam to condition the oil by reducing its viscosity, (2) injecting solvent to recover remaining oil through chemical diffusion and gravity segregation, and (3) injecting steam or hot water to retrieve the trapped solvents with remaining oil. According to experimental observations and numerical studies, trapped solvents could be recovered up to 80–85% with 85–90% of the original oil in place.

In tight matrixes, such as shale and tight sandstone reservoirs, injecting steam could be inefficient in some cases, owing to the great restriction of steam propagation through the reservoir. This restriction leads to considerable volumes of the injected steam condensing in near-wellbore regions because of the ultra-low rock permeabilities. Since the water density is higher than the steam density, the mobility of the hot water through the reservoir is reduced by the tightness of the rocks thus resulting in an enormous heat loss before reaching the bottomhole of the well. Similarly, injecting liquid hydrocarbon solvents into tight reservoirs is a challenging application as the low permeability acts as a barrier against the movement of liquid solvents in the reservoir. Such phenomenon decreases the contact of solvents with the targeted oil; as a result, the reduction of oil viscosity, through solvent diffusion, would take place with only a small volume of the oil in the reservoir.

An alternative option is to inject gas solvents (methane, propane, CO_2_), since their low densities and viscosities help in enhancing the propagation of solvents in the matrix. Propane injection has gained substantial attention over the last decade as one of the more efficient EOR applications to recover heavy oil in unconventional reservoirs. Favorable physical properties of propane have made the usage effective and highly desirable for tight matrixes. According to Nagarajan et al.^5^, one of the significant benefits of injecting propane in the tight Bakken reservoirs was that it contacted greater volumes of oil in the reservoir due to its first-contact miscible pressure (650 psi) at the reservoir temperature. Thus, injecting the gas improved the mobility of larger oil volumes by reducing their viscosities.

The phase-change is controlled not only by pressure and temperature, but also capillary and interfacial characteristics as the porous media becomes tighter. This phenomenon leads the vapour pressures and boiling points to deviate from the bulk conditions and this process is controlled by the size of the pores and wettability conditions. To model hybrid or sole-solvent injection precisely under non-isobaric and non-isothermal conditions, actual phase-change behaviours of fluids in various porous media should be well understood through experimental and theoretical investigations. Studying vapour pressure alteration of propane in extended tight rocks, such as shales, was experimentally performed by Zhong et al.^6^ by using nanofluidic chips featured with silicon nanochannels. The chips had various sizes of channels ranging from 20 μm to 8 ${\text{nm}}$. Condensation of propane within the confined channels was observed under a range of pressure ($\sim 0.6 - 2.3 \,{\text{MPa}}$) and temperature (286.15–339.15 °K). The study also aimed to validate theoretical modelling (Kelvin equation) by comparing calculated results with the experimental outcomes. Vapour pressures, obtained from the Kelvin equation, were closely matched to the experimental outcomes even in extended confined channels ($\sim 8 \,{\text{nm}}$) making the equation applicable in modelling capillary condensation in silica-glass media. Vapour pressure alteration of water in nanochannels was inspected by Tsukahara et al.^7^ using a nanoscale chip. The nanofluidic chip consisted of microchannels (10 μm deep) and nanoscale channels with a depth range of 90–370 ${\text{nm}}$. The experiments demonstrated the reduction of water vapour pressure with the decrease of medium size. Additionally, computed vapour pressures from the Kelvin equation were relatively similar to those observed in the experiments; therefore, applicability of the Kelvin equation in predicting vapour pressures within extreme confined fused-silica glass media remains valid.

Recent studies show that the accuracy of the Kelvin equation declines in nanopores smaller than 8 ${\text{nm}}$. Wang et al.^8^ investigated the precision of computed phase-change pressures by the Kelvin equation and equation-of-state-with-capillary-pressure (EOS-P_cap_) in extended confined pores. Quantitatively, the study demonstrated that overestimation and underestimation issues were noticed with the Kelvin equation and EOS-P_cap_ when predicting condensation and evaporation pressures of propane in pores below 8 ${\text{nm}}$, comparing with the density function theory (DFT) predicted outcomes. However, reasonable accuracy of vapour pressure prediction could be achieved with the thermodynamical models when the pore size is above 8 ${\text{nm}}$.

The curvature effect on fluids increases as capillary size becomes tighter due to the change of interfacial properties, such as surface tension and pressure difference at the liquid–gas interface. When the pore becomes smaller, the pore-fluid interaction begins to display an influence on the phase-alteration nature of confined fluids. In a nanopore, due to the limited number of molecules, a large percentage of molecules are absorbed by the pore wall, and condensation/vaporization behaviours begin to alter from bulk conditions when the medium gets tighter than 100 ${\text{nm}}$^9^. Fluids in confined spaces could gain distinctive properties, such as higher viscosity and slower motion of molecules, which could be the reason behind the phenomenon of shifted vapour pressures^7^. Several investigations were performed to understand the deviation of phase-change behaviour in extreme tight channels featured with nanoscale pore throats^10^. The majority of these studies were conducted using microfluidic and nanofluidic chips made of silica glass. Per the Kelvin equation, the alteration of vapour pressure in confined pores depends on capillary size, surface tension, and contact angle between the solid surface and liquid. These liquid–solid properties, including molecular absorption of the surface, might change noticeably when the solid material is altered. This paper aims to study the phase-change behaviour of propane in various capillary media starting from Hele–Shaw glass cells to real core samples with different permeabilities, porosities, and pore throat sizes. Moreover, microfluidic chips with uniform and non-uniform properties (grain and pore throat sizes), representing various pore sizes, were utilized to obtain a clearer picture of propane phase alteration in porous media using visual support. One of the main targets in this investigation was to inspect the effect of surface properties on propane’s vaporization in confined spaces and compare the experimental outcomes with the computed saturation pressures, computed by the Kelvin equation and Peng–Robinson equation-of-state (EOS). Comparative analysis of the outcomes obtained from glass microfluidic experiments and rock samples provided new insight into the pore scale thermodynamics of the solvents to be used in further computational studies to improve the accuracy of performance prediction.

## Background

The Kelvin equation^11^ is a theoretical modelling approach that describes the influence of curvature radius at vapour-liquid interface on saturation pressures. The general Kelvin equation can be expressed as

1 $$RT{\text{ ln}}_{ } \left( {\frac{{P_{v} }}{{P_{\infty } }}} \right) = - \frac{{2\sigma^{LV} v^{L} }}{r} + v^{L} \left( {P_{v} - P_{\infty } } \right),$$

where $T$ is the fluid temperature, $R$ is the universal gas constant, $r$ is the droplet (or capillary) radius, $v^{L}$ is the molar volume of the liquid, $\sigma^{LV}$ is the vapour-liquid interfacial tension, $P_{\infty }$ is the vapour pressure at the flat surface, and $P_{v}$ is the vapour pressure at the curved interface. The term $\left( {P_{v} - P_{\infty } } \right)$ on the right side of Eq. (1) can be neglected owing to its small value comparing to the first term ($- 2\sigma^{LV} v^{L} \backslash r$)^12^. Hence, the approximated form of the equation is as follows:

2 $$RT{\text{ ln}}_{ } \left( {\frac{{P_{v} }}{{P_{\infty } }}} \right) = - \frac{{2\sigma^{LV} v^{L} }}{r}.$$

When the medium is liquid wet (concave curvature), the vapour pressure of the liquid reduces along with the reduction of pore size thus leading the vapour pressure at confined spaces ($P_{v}$) to be lower than the vapour pressure at the flat surface ($P_{\infty }$) or bulk condition. One of the major limitations of the Kelvin equation is that it is not applicable for computing the shift of vapour pressure of multicomponent fluids in hydrocarbon reservoirs, due to their complexity. In petroleum industries, cubic EOS is commonly used to estimate the phase behaviour of reservoir fluids which helps in forecasting approximated oil recoveries^13^. Peng–Robinson EOS^14^ is considered one of the more common models in reservoir engineering to predict the phase-change behaviour of hydrocarbon mixtures in the reservoir. For a single-component fluid, PR-EOS can be expressed as

3 $$P = \frac{RT}{{V_{m} - b}} - \frac{a \alpha }{{V_{m}^{2} + 2bV_{m} - b^{2} }},$$

where $R$ is the universal gas constant, $T$ is the fluid temperature, $a {\text{and}} b$ are constant parameters, $\alpha$ is a temperature dependence function which is related to the reduced temperature and acentric factor, and $V_{m}$ is the molar volume. Later on, the accuracy of Redlich–Kwong (RK)^15^ and Peng–Robinson EOS’s was improved in predicting phase behaviour of complex hydrocarbon multicomponent mixtures and liquid densities. Le Guennec et al.^16^ developed the improved versions of PR and RK cubic EOS by introducing a consistent $\alpha$-function which ensures a precise vapour-liquid equilibrium (VLE) calculation with multicomponent fluids and provides accurate extrapolations in areas above critical points. The volume translation was also considered to achieve correct saturated liquid volumes which closely match with the experimental outcomes. Pina-Martinez et al.^17^ proposed an updated version of Soave $\alpha$-function for PR and RK equation of state. The corrections made in both cubic EOS were constructed based on a wide range of compounds (1721 pure compounds) from various chemical families. Such improvements had impacts on enhancing the reproduction of vapour pressures, calculated by the PR EOS. Considerable enhancements were noticed in systems with heavy molecules.

Peng–Robinson EOS and Redlich–Kwong EOS are widely used cubic EOS models in petroleum applications, owing to their more accurate critical compressibility factors ($Z_{C}$) that are closer to experimentally measured values^13^. Though, one of the drawbacks of these cubic EOS models is that they do not consider the confinement effect which causes them to lose some of their accuracies in situations where tight rock media is involved. Reservoir rocks are heterogeneous systems which consist of pores with a varied range of sizes. Generally, extended small channels (< 1000 nm) commonly exist in tight rocks, such as tight sandstone and shale. Thermodynamically, the phase-change behavior of fluids begins to be influenced by the medium sizes when they are smaller than 1000 nm, as stated by the Kelvin equation. Based on our pore scale distribution analysis, micropores ($< 2 \,{\text{nm}}$) and mesopores (2–50 nm) do exist in permeable rocks with minor pore volumes––which were estimated to be less than 5% of the total pore volume per mass unit. The investigation in this paper focused on observing the phase-alteration of propane under various temperatures in different rock types and compared the measured vapour pressured with computed phase-change pressures from the Kelvin equation and Peng–Robinson EOS^14^.

## Statement of the problem and objectives

In heavy-oil recovery applications, injecting solvents with or without steam under variable pressure and temperature could lead to considerable phase alteration. The thermodynamics of injected fluids in porous media play a critical role in controlling the performance of hybrid and cold-solvent injection. Phase alteration during the process could control the distribution of injected fluids in the reservoir as well as their flow dynamics and eventually, oil recovery. Similarly, solvent retrieval process (SRP) highly depends on solvent thermodynamics in the reservoir. As a result, comprehending the phase-change behaviour of injected fluids is important in choosing the appropriate application conditions (such as pressure and temperature) while maximizing oil recovery and solvent retrieval. SRP is a critical part of the whole process and has an impact in minimizing the overall operational cost of hybrid (steam-solvent) applications. The phase change mechanism should be well understood during this process as it directly affects the oil recovery (during injection) and solvent retrieval (during depletion), and the phase behaviour in capillary medium is different from the bulk conditions of which are applied in classical PVT tests and studies.

A similar phenomenon is encountered in oil (heavy-oil, light, oil, and condensate) recovery from unconventional (shales, tight sands) reservoirs in which the most common application suggested is solvent gas (hydrocarbon gases or CO_2_). The gases injected (in the form of huff-and-puff) after fracking diffuse into the rock matrix and reproduce with oil during the depletion stage. The recovery of oil and solvent retrieval are both controlled by the thermodynamics (mainly the phase change of the solvent and oil). It is well-known that the phase change conditions in capillary medium differ from the bulk conditions and this cannot be captured easily using standard PVT analyses. Per the Young–Laplace equation ($$\Delta P = 2\gamma /r)$$, the curvature radius ($$r)$$ has an effect on surface tension ($$\gamma$$) when it decreases to microscales or nanoscales^7^. The Kelvin equation demonstrates the relationship between vapour pressures in capillary and bulk conditions $$\left( {P_{r} = P_{\infty } \exp \left[ {\frac{{2 \sigma v^{L} }}{r R T}} \right]} \right)$$. According to this equation, the vapour pressure of fluids becomes lower than those in bulk scenarios when medium sizes are tighter due to the change of surface tension, pressure drop at the interface, and contact angle. As a result, fluids in highly confined spaces tend to have higher viscosities and capillary pressures.

The objective of this paper was to experimentally investigate the vapour pressure of propane in different capillary models and compare the outcomes with computed vapour pressures from the Kelvin equation and Peng Robinson EOS. The vapour and condensation pressures of propane were measured using Hele–Shaw cells, capillary tubes, and homogenous/heterogeneous micromodels with various pore throat and grain sizes. As a more realistic porous media representation, rock samples such as Berea sandstones, Indiana limestone, tight sandstone, and shale were also considered, and the vapour pressure of propane was measured in those samples to obtain a wider perspective of how the vaporization of propane occurs in various capillary media with dissimilar surface properties and porous structures. Furthermore, the results with rock samples were compared with outcomes obtained from Hele–Shaw and micromodel glass chips, including the computed saturation pressures obtained by the thermodynamical models.

## Experimental study

Observations of propane’s vaporization pressures were performed by using several glass chips and rock samples. The experiments were initiated with Hele–Shaw glass cells of 0.13 and 0.04 mm gap thicknesses. Although the Hele–Shaw cells represent only a simple tight system with smooth and liquid-wet inner surface, they can be useful in providing a clear visualization of bubbles generation of fluids in different pressures that could be difficult to visualize in microfluidic chips. Then, vapour pressures of propane were inspected in several types of micromodels with uniform and non-uniform properties such as porosity, permeability, and pore throat/grain size.

### Hele–Shaw glass cells

**Figure 1:**
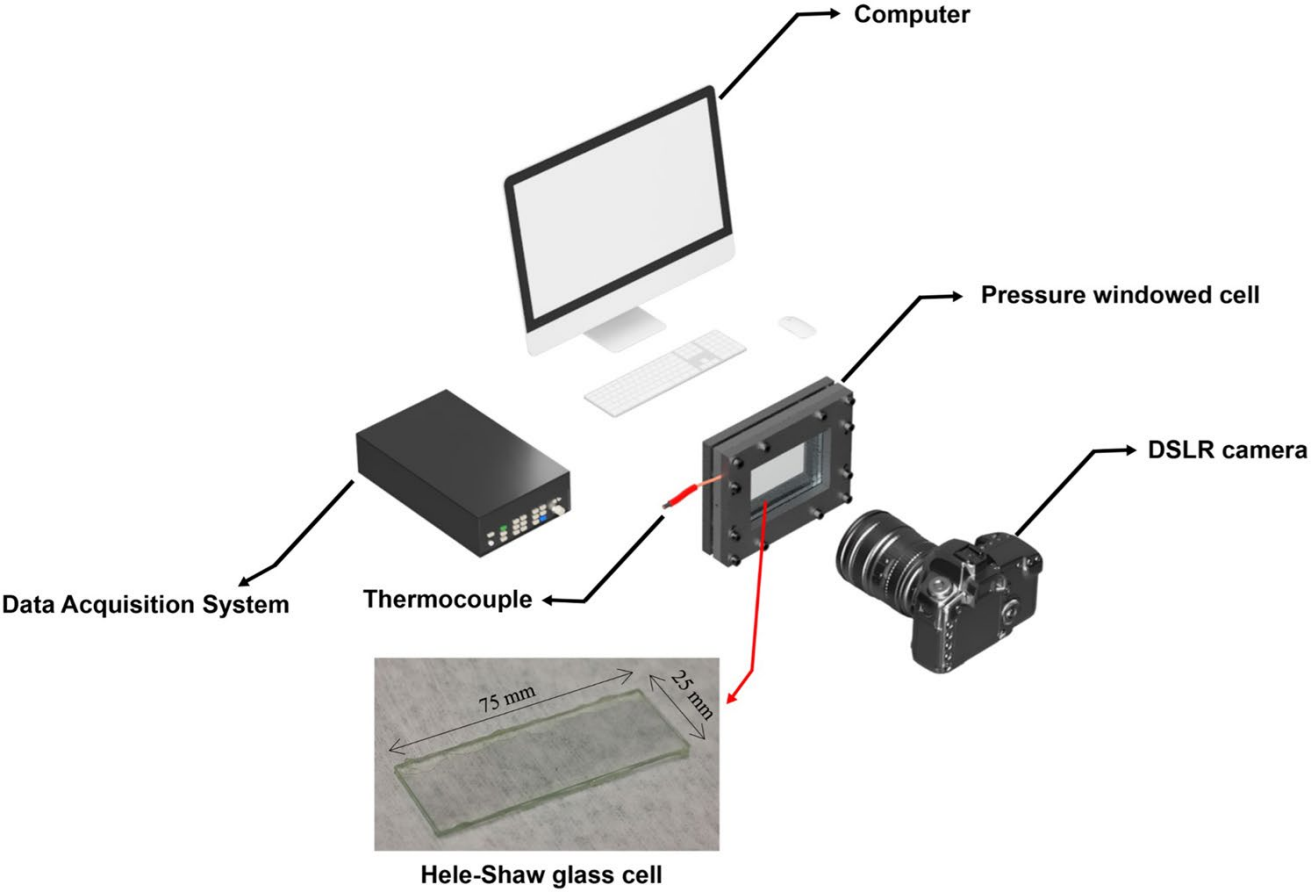
Experimental setup used in Hele–Shaw experiments.

Hele–Shaw cells basically consist of a pair of thin rectangular glass plates with an empty gap in between. The glass cells were made with two main gap thicknesses: (1) 0.04 mm and (2) 0.13 mm. Mainly, the purpose of starting our investigation with glass cells was to get a clear exposure of propane’s bubbles formation in pressure depletion stage under constant temperature ($\approx 20\,^{ \circ } {\text{C}}$, 293.15 K). In all Hele–Shaw cells, the inner glass surfaces were propane-wet during condensation. Figure 1 presents the Hele–Shaw glass cell used in our experiments. The experimental setup consisted of a DSLR camera, pressure and temperature measurement device, LED light source, thermocouple, pressure transducer, ISCO syringe pump, and pressure windowed cell. Figure 1 illustrates some of the equipment used in experiments with Hele–Shaw cells.

#### Procedure

To pressurize the Hele–Shaw glass models, they were placed in the pressure windowed cell. The pressure cell was featured with plexi-glass windows which allowed clear visualization through the cell. By using an ISCO syringe pump, the cell was pressurized from a starting pressure ($70 \,{\text{psi}}$) to 150 psi––which is above the propane vapour pressure (in Edmonton, Alberta, Canada) and is approximately 115 psi in atmospheric temperature ($\approx 20\,^{ \circ } {\text{C}}$, 293.15 K). The pressure range was selected based on the pressure limitation of the windowed cell that could withstand a maximum pressure of 160 psi. Both the Hele–Shaw glass cell and pressure windowed cell were vacuumed for a period of time to remove the trapped air in the system. We aimed to study the vaporization and condensation of propane; hence, the pump was programmed to build up and deplete the pressure at a rate of 5–7 psi/min within 10 min. Meanwhile, a continuous video was taken with the DSLR camera during the process. Additionally, the pressure and temperature in the pressure cell were recorded constantly by the measurement device every 2 s.

#### Results and discussion

**Figure 2:**
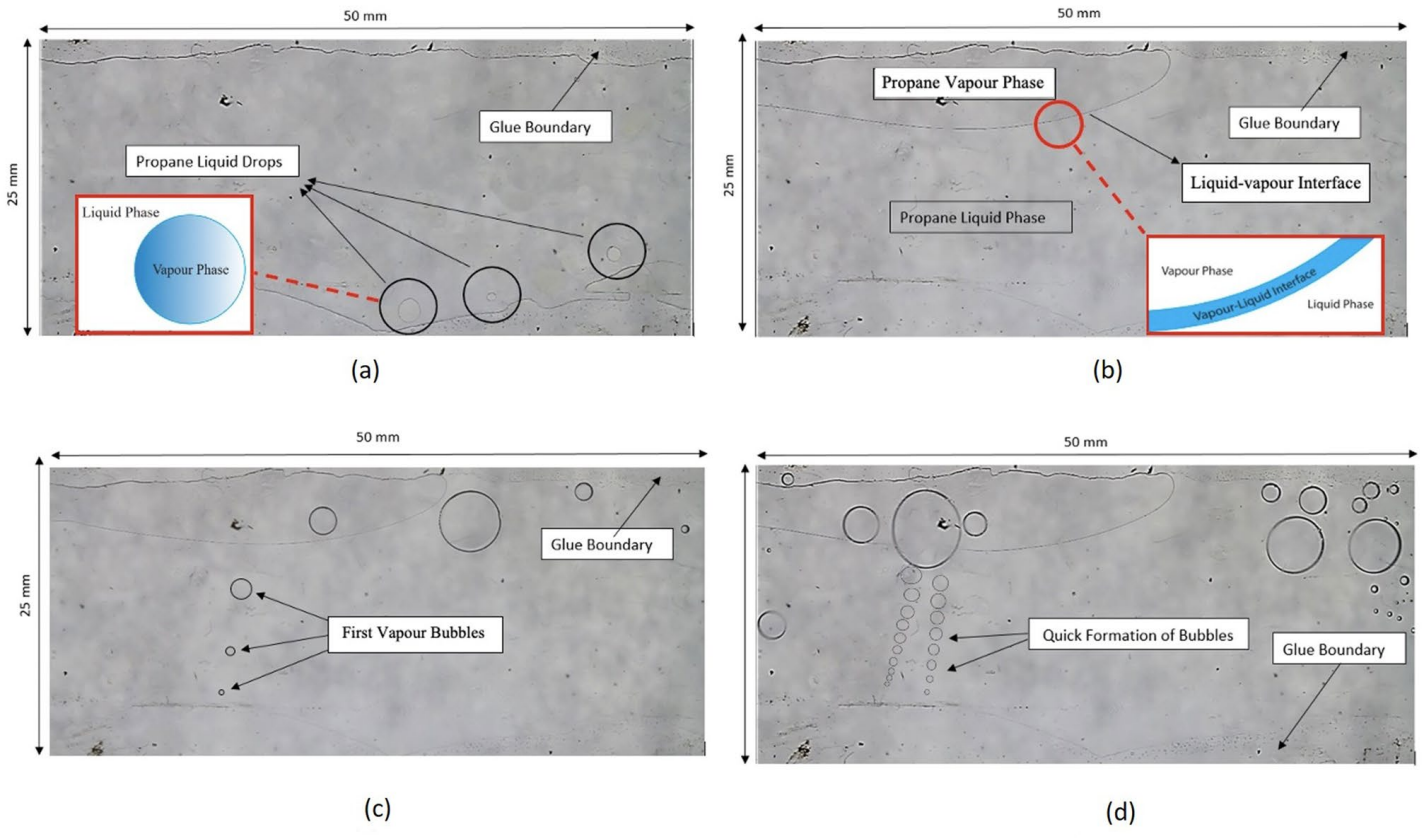
(**a**) Considerable phase change at 121.2 psi; (**b**) dew point stage at 118.5 psi; (**c**) bubble point stage at 116.6 psi; (**d**) quick formation of bubbles stage at 113.7 psi.

**Figure 3:**
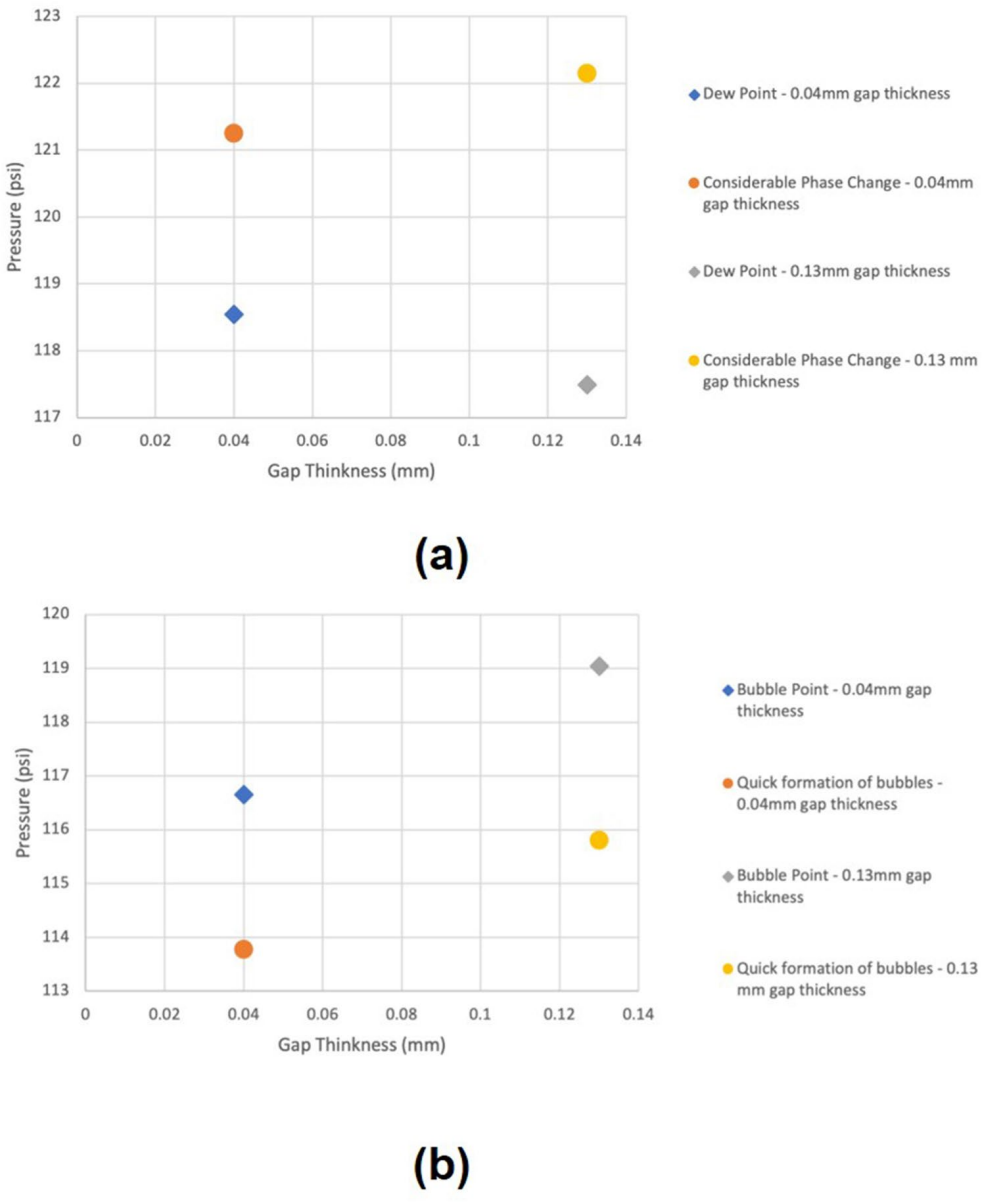
(**a**) Pressure at each stage in 0.04 and 0.13 mm gap thickness during pressure build-up process; (**b**) pressure in 0.04 and 0.13 mm gap thickness during pressure depletion process.

As mentioned previously, Hele–Shaw cells provide a clearer visualization of the nucleation stage, unlike microfluidic chips and rocks. Using the glass cells could bring several limitations (flat liquid–solid interface) with it which might not act as a good representation of real reservoir conditions. However, they could be useful in illustrating the phenomena under the simplest conditions. It was expected that the vapour and condensation pressure of propane in the glass cell would be relatively close to bulk pressures since their gap thicknesses were not tight enough to create effective changes in surface tension ($\gamma$) and contact angle ($\cos \theta$). During the condensation process, propane went through two main stages: (1) dew point and (2) considerable phase change. In the vaporization process, two stages were considered: (1) bubble point and (2) quick formation of bubbles. In the Hele–Shaw cell with 0.04 mm gap thickness, the first propane liquid drops took place at 118.5 psi as shown in Fig. 2a. A considerable phase change initiated in the cell at a pressure of 121.2 psi (Fig. 2b). In the pressure depletion stage, the first propane bubbles generation took place at 116.6 psi as illustrated in Fig. 2c. A quick formation of propane bubbles began in the glass cell at 113.7 psi (Fig. 2d). Figure 3 show the pressures in 0.04 mm and 0.13 mm gap spaces during the build-up and depletion processes.

### Microfluidic chips

Compared to Hele–Shaw glass cells, micromodels offer a better representation of porous media in terms of size and shape of the pores. Three categories of microfluidic chips were used: (a) capillary tube model, (b) homogenous micro model, and (c) heterogeneous micro model. Capillary tubes represented straight silica-glass pore throats with various sizes ranging from 40 to 1 µm (µm). Homogenous micromodels were designed with uniform grain and pore throat sizes. In our experiments, two homogeneous models with different properties were utilized: (1) a microfluidic model with uniform properties of 0.11 mm pore diameter and 0.01 mm pore throat and (2) a model with uniform properties of 0.21 mm pore diameter and 0.01 mm pore throat. The heterogeneous microfluidic chip had a porous structure closer to real rocks with an average pore throat of 142.5 µm.

#### Procedure

To pressurize the microfluidic chips, they were placed in the pressure windowed cell, and using an ISCO syringe pump, the cell pressure was increased from $70 \,{\text{psi}}$ to 150 psi which is above the propane vapour pressure (115 psi at $20\,^{ \circ } {\text{C}}$, 293.15 K). The pressure range was selected based on the pressure limitation of the windowed cell that could withstand a maximum pressure of 160 psi. The entire system was vacuumed for a period of time to remove the trapped air inside the windowed cell and silicate glass micromodel. The pump was programmed to build up and deplete the pressure at a rate of 5–7 psi/min within 10 min and a continuous video was taken by the DSLR camera during the process. Additionally, the pressure and temperature in the system were recorded constantly by the measurement device every 2 s.

#### Results and discussions

**Figure 4:**
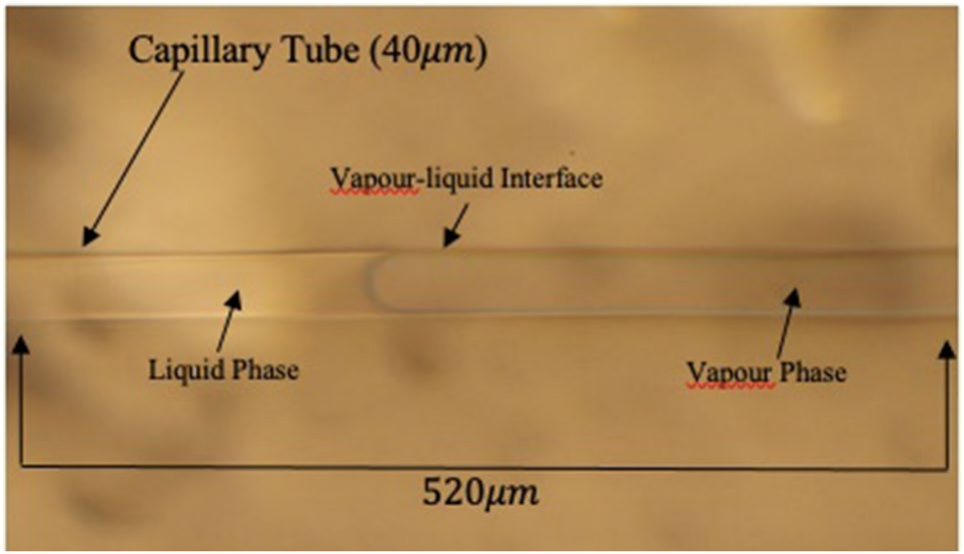
Propane vaporization in the 40 μm capillary tube.

The micromodel experiments were initiated with capillary-tube models featured with five sizes: (a) 40 µm, (b) 20 µm, (c) 10 µm, (d) 5 µm, and (e) 1 µm. Through capillary-tube experiments, it was noticed that liquid propane wets the inner surfaces of the tubes during the condensation stage which makes these models act as propane-wet capillary media. In the 40 µm tube, during the pressure depletion process, vaporization of propane took place in the tube at 116.1 psi, as shown in Fig. 4. Table 1 shows the vapour and condensation pressures of propane in various sizes of capillary tube during the depletion processes. The phase-change pressures were comparable with the pressure values recorded in the bulk conditions because of the size of tubes which had almost no effect on the vaporization and condensation behaviour. Medium (pore) sizes have influences on the phase-alteration behaviour of fluids when they are 100 nm or smaller^9^.

**Table 1:** Vapour and condensation pressures of propane in several capillary-tube sizes.

	Vapour pressure (psi)	Condensation pressure (psi)
40 µm Capillary tube	116.1	118.2
20 µm Capillary tube	113	121.3
10 µm Capillary tube	120.1	116.6
5 µm Capillary tube	116.1	123
1 µm Capillary tube	121.3	121.5

**Figure 5:**
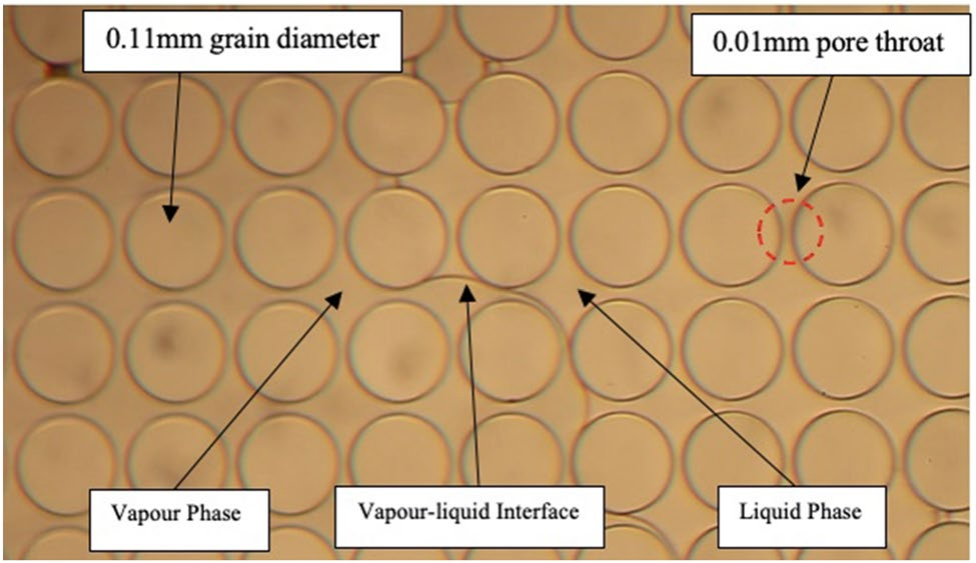
Propane vaporization in the homogenous micromodel (0.11 mm grain diameter and 0.01 mm pore throat).

**Figure 6:**
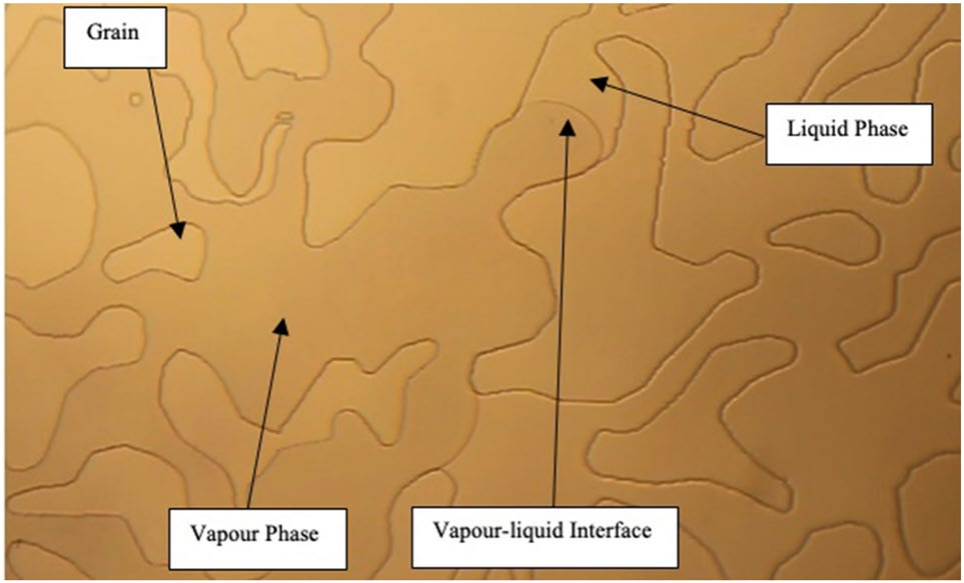
Propane vaporization in the heterogeneous micromodel (average pore throat size of 142.5 μm).

In microfluidic homogenous chips, phase-change pressures were relatively similar to those observed with capillary tube models. In the homogenous model (0.11 mm grain diameter and 0.01 mm pore throat), propane vaporization began at 115.3 psi, as illustrated in Fig. 5. Comparably, propane phase change took place in the heterogonous microfluidic model at 118.8 psi (Fig. 6). Table 2 presents the propane vapour and condensation pressures in homogenous and heterogonous models. As observed in the Hele–Shaw experiments, the pore throat sizes in the microfluidic models were not confined enough to alter the vaporization and condensation behaviour. The phase-change pressures of propane in the homogeneous and heterogeneous model were comparable with the bulk values.

**Table 2:** Vapour and condensation pressures in homogenous and heterogonous microfluidic chips.

	Vapour pressure (psi)	Condensation pressure (psi)
Homogenous model (0.11 mm grain diameter and 0.01 mm pore throat)	115.3	119
Homogenous model (0.21 mm grain diameter and 0.01 mm pore throat)	117	118
Heterogeneous model (average pore throat size of 142.7 µm)	118.8	119.1

### Rock porous media

To visualize the phase change of propane in more realistic porous media, vapour pressure alteration was examined in Berea sandstone, Indiana limestone, tight sandstone, and shale rock samples. Using real rock samples provided an advantage of testing the impact of surface characteristics and porous media structure on the vapour pressure at different surrounding temperatures, ranging from 0 °C (273.15 K) to 40 °C (313.15 K). Due to their different surface properties and pore sizes, it was expected that the vapour pressure of propane might alter, comparing with those observed in the Hele–Shaw cells and microfluidic silica-glass chips. The average permeability defers from one rock to another, depending on the rock nature. Permeabilities, in some cases, could reflect an approximated insight of the pore sizes that a rock might have. The Winland equation^18^ is one of the approaches that can be utilized to estimate the average pore size of a rock by knowing its permeability and porosity, and it is commonly used by the petroleum industry^19^. The equation, however, computes pore sizes empirically which makes its outcomes highly approximated with a considerable lack of accuracy in certain cases. The pore size distribution analysis assisted us to experimentally measure the actual pore volumes of micro and meso channels in the rock surface. The analysis also allowed us to find the volume percentage of pores tighter than 1000 nm in each rock.

#### Pore size distribution analysis (PSDA)

**Figure 7:**
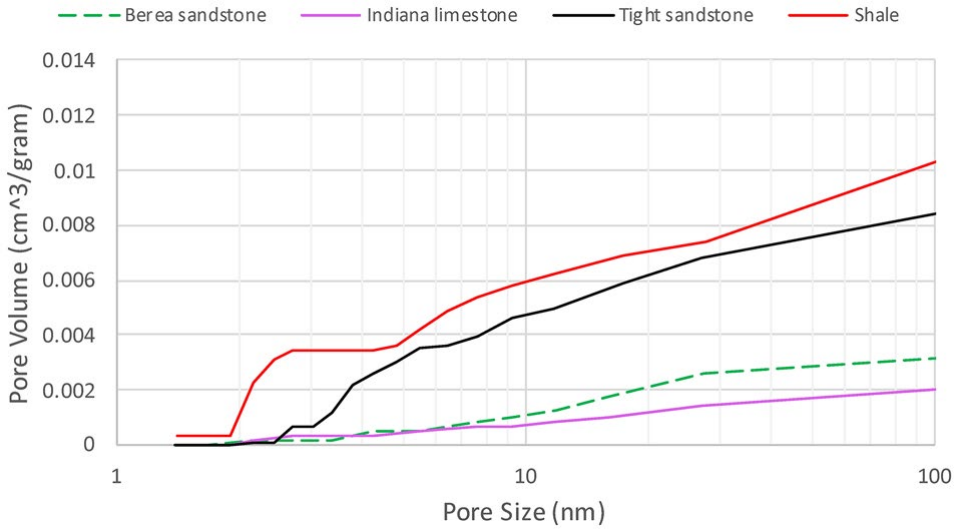
Change of pore volumes of various pore diameters, ranging between 1 and 100 nm, based on nitrogen desorption^20^.

PSDA was performed by a Brunauer–Emmett–Teller (BET) surface area analyser; the surface area is the available pore area for nitrogen adsorption. The BET surface area, average pore diameter, and total pore volume were evaluated by physisorption of nitrogen in rock porous media. Despite the high permeabilities and porosities that permeable rocks have, inconsiderable pore volumes of nanopores might exist, which, in theory, could alter the phase-change behaviour of fluids located within these tight pores. The investigation evidenced the existence of micro and meso pores in the permeable rocks (sandstone and limestone). Table 3 shows the measured permeability, density, and median pore size of channels below 1000 nm, and volume percentage of pores smaller than 1000 nm in sandstone, limestone, tight sandstone, and shale. The volume percentages were estimated based on the mass unit (gram). Figure 7 shows the deviation of pore volumes of different pore sizes in each rock type. In shale and tight sandstone, the pore volumes of pores, with a size range of 3–10 nm, are higher than what is observed with sandstone and limestone. Also, larger pore volumes in mesopores (2–50 nm) are detected in shale and tight sandstone, compared with sandstone and limestone.

**Table 3:** Average permeability, rock density, and pore volume percentages of various rock types^20^.

Rock type	Average permeability (millidarcy)	Density (${\text{kg}}\,{\text{ m}}^{ - 3}$)	Median pore size of pores smaller than 1000 nm	Volume percentage of pores smaller than 1000 nm (%)
Berea sandstone	274	2129	350	4.4
Indiana limestone	30	2246	470	4.6
Tight sandstone	0.1	2400	300	38.2
Shale	< 0.01	2200	125	94.3

The analysis showed that minor volume percentages (< 5%) of confined pores (< 1000 nm) could be observed in sandstone and limestone despite their high permeabilities. Due to the tight nature, pore volumes of constrained channels in tight sandstone and shale are significantly higher, as presented above in Table 3. Having higher pore volumes of nanopores in tight sandstone and shale would increase the volume of inner fluid that could vaporize at pressures or temperatures different from the bulk conditions. On the other hand, in sandstone and limestone, lower inner fluid volumes would vaporize differently from the bulk values owing to their considerably lower nanopore (< 1000 nm) volumes. Nonetheless, at reservoir scales, the shift of saturation pressure in permeable rocks could be a noticeable impact on the simulated oil recovery or history matching.

#### Experimental setup

**Figure 8:**
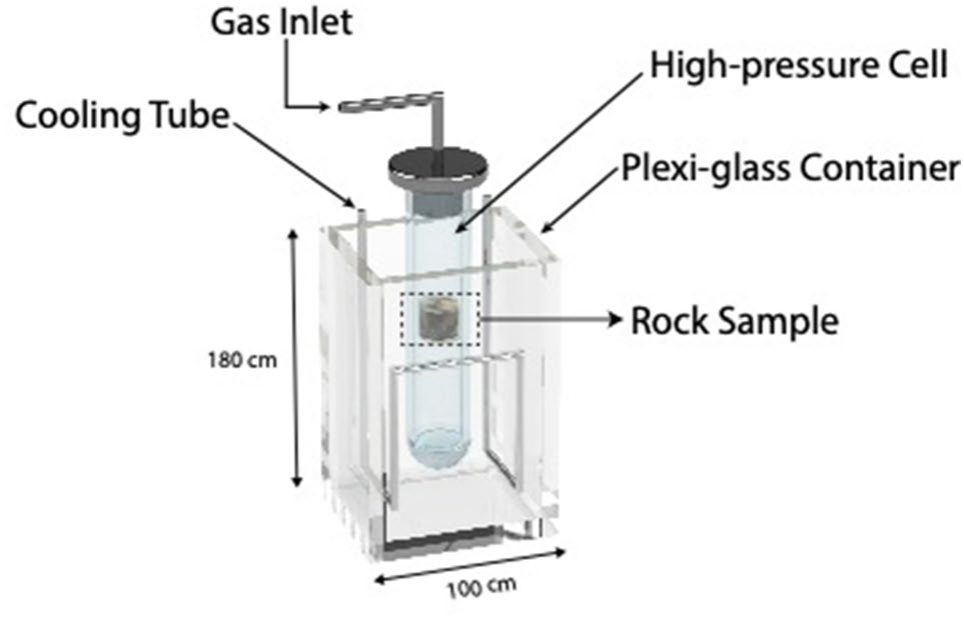
Cooling liquid bath used to reduce the rock’s temperature below ambient temperature (20 °C, 293.15 °K). The cooling liquid (water) was placed in the plexi-glass container and cooled gradually by the cooling metal tube.

**Figure 9:**
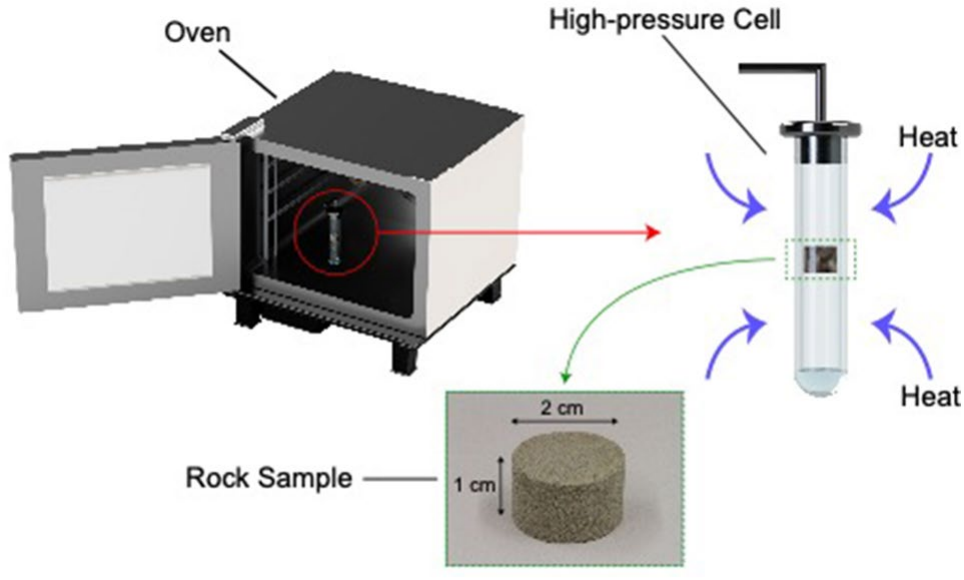
Constant-temperature oven utilized to increase the rock’s temperature above the ambient temperature. The oven ensured a uniform heat migration to the system and a homogeneous temperature distribution around the rock.

The vapour pressure of propane was studied in various reservoir rocks at different temperatures, including temperatures lower than the ambient temperature ($\approx 20\,^{ \circ } {\text{C}}$, 293.15 K). A special borosilicate-glass cell was utilized to pressurize the rock samples above the propane condensation pressure. A cooling liquid bath (Fig. 8) was used to reduce the temperature of rocks and precisely stabilize it throughout the experiment. The liquid bath (water—H_2_O) temperature was decreased by pumping a liquid coolant through the metal tube at desired temperatures. At freezing temperatures ($\le 0\,^{ \circ } {\text{C}}$), water was freezing at areas around the metal tube. However, due to the naturally slow freezing process, water remained in its liquid form around the glass cell during the experiments. With a constant-temperature oven, the glass cell and rock samples were heated at various temperatures above the ambient temperature (Fig. 9). Using an ISCO syringe pump allowed us to pressurize the system and then deplete the pressure accurately at a specific depletion rate. The temperature inside the glass cell was measured and recorded continually by a measurement device.

#### Procedure

Ensuring a high purity of gas within the system was a critical part of our experiments. Including the rock samples, the whole system was vacuumed thoroughly to remove the trapped air and achieve the maximum purity of propane inside the system. After the vacuuming process, the glass cell was pressurized with propane (purity of 99.95%) with the assistance of an ISCO pump. To guarantee a complete condensation, propane was pressurized 20 psi more than its saturation pressure. Generally, the vapour pressure of gases increases as their temperatures rise. The glass cell was limited with a maximum pressure of 220 psi. Therefore, the experiments were restricted with a maximum temperature of 40 °C (313.15 K) since the vapour pressure of propane, at bulk condition and 40 °C, is 200 psi. Then, the overall pressure in the system was depleted at a constant rate of 0.6 psi per minute. The vaporization of propane in different rocks was studied at several temperatures which were 0 °C (273.15 K), 10 °C (283.15 K), 20 °C (293.15 K), 30 °C (303.15 K), and 40 °C (313.15 K).

#### Results and discussion

**Figure 10:**
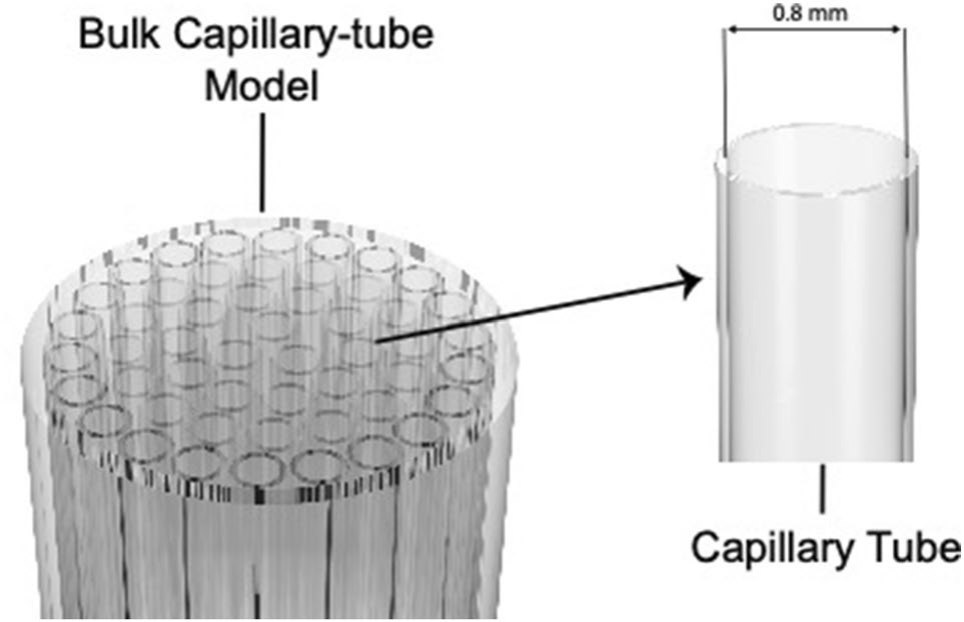
Silica-glass bulk model consisting of capillary tubes with constant diameters of 0.8 mm.

**Figure 11:**
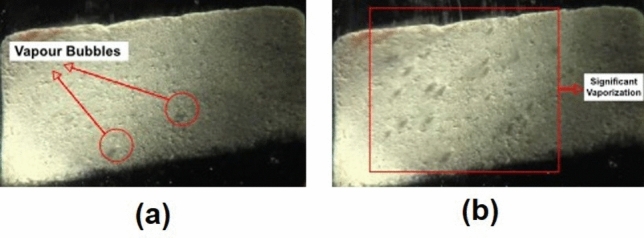
(**a**) Initiation of vapour-phase (nucleation) in Berea sandstone; (**b**) significant propane vaporization in Berea sandstone.

The investigation of propane phase-change behaviour in various reservoir rocks focused on two main stages of vaporizations: (a) initiation of vapour-phase formation (nucleation), and (b) significant vapour generation. Due to the heterogeneity of pore interconnectivity, the movement of vapour bubbles within the rocks could slightly vary in every experiment; consequently, the temperature at which the bubbles appearing on the rock surface could vaguely change between the trials. Hence, with each rock type, repeatability was the main key to achieve representative outcomes by averaging the measured vapour pressures. Initially, the propane vapour pressure was investigated in bulk conditions using bulk models. The models consist of 0.8 mm silica-glass tubes (Fig. 10); thermodynamically, their diameters should not impact the phase-change behaviour of propane. With the bulk model, the outcomes were relatively similar to the handbook values, as shown in Figs. 13 and 14. In sandstone and limestone, the vapour pressure of propane was noticeably lower than the bulk values, due to the existence of nanopores (< 1000 nm). For instance, Fig. 11 presents the two stages of phase change in Berea sandstone at 30 °C (303.15 °K). The bulk vapour pressure of propane at temperature of 30 °C is 170 psi. In sandstone, the nucleation took place at 159 psi, as shown in Fig. 11a. A significant phase alteration was observed at a pressure of 163 psi (Fig. 11b).

### Quantitative analysis

**Figure 12:**
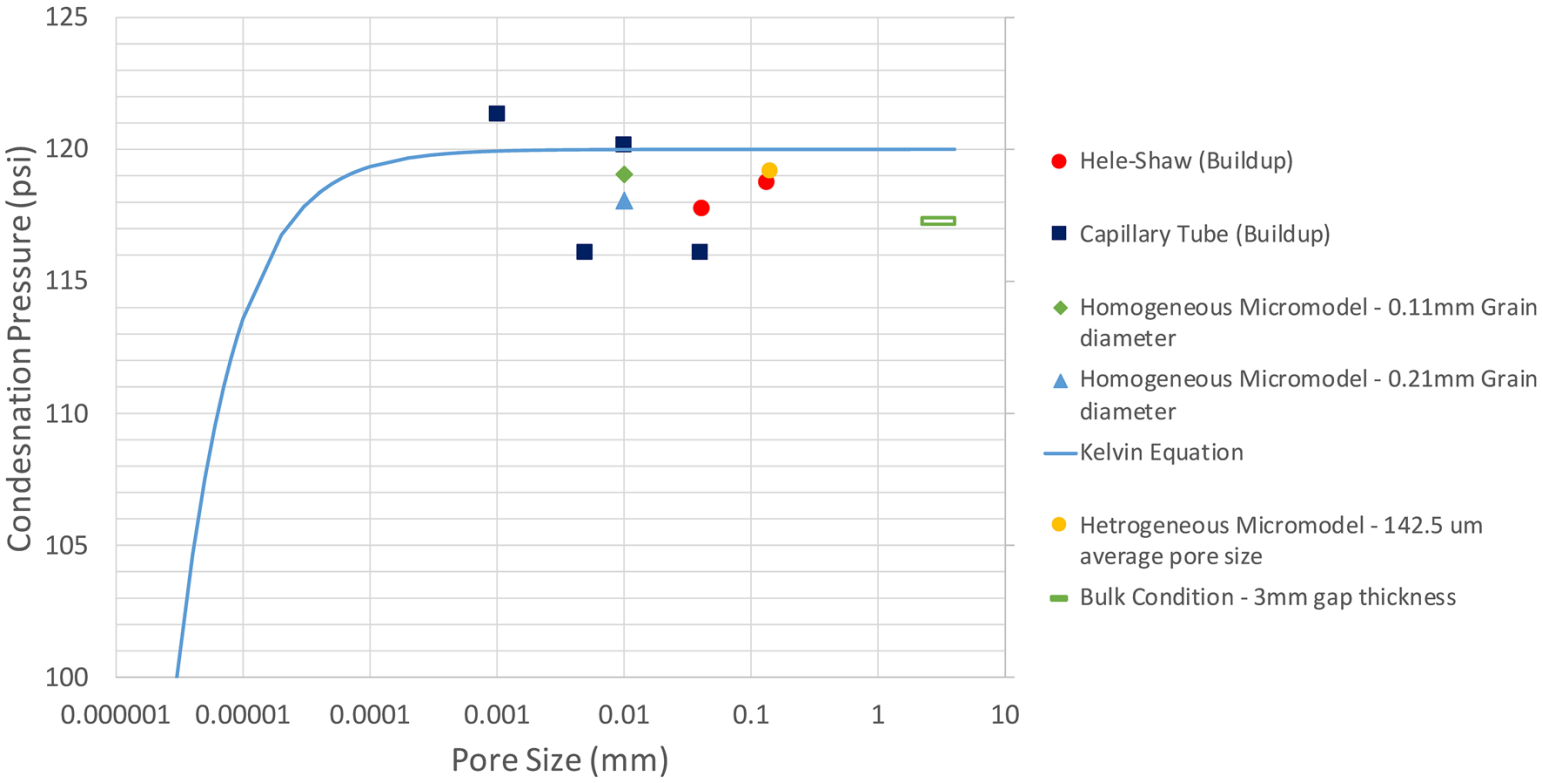
Condensation pressures of propane in Hele–Shaw cells and micromodels during the pressure build-up process. Each point for the bulk condition represents the average of 3 trials, and each point for the Hele–Shaw cell and microfluidic chip represents the average of 2 trials. All the pressure values in this figure were measured at 20 °C (293.15 K).

**Figure 13:**
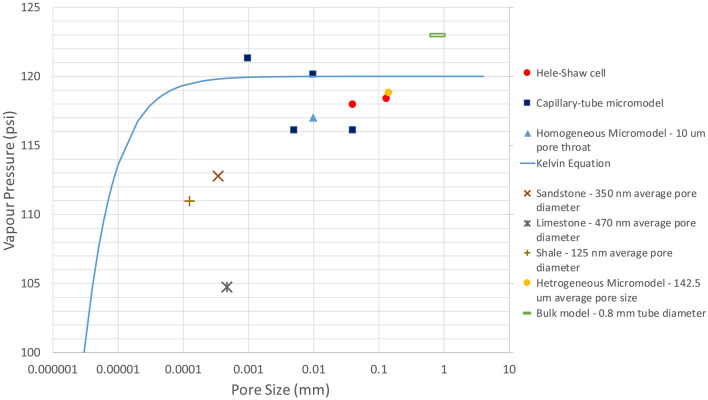
Measurement of propane vapour pressure in Hele–Shaw cells, micromodels, and rock samples. Each point for sandstone, limestone, and shale represents the average of 3 experiments (3 trials with 3 core samples from identical reservoir rock blocks). Each point for the bulk condition represents the average of 3 trials, and each point for the Hele–Shaw cell and microfluidic chip represents the average of 2 trials. All the pressure values in this figure were measured at 20 °C (293.15 K).

Per the Kelvin equation, vapour pressure could be altered by medium size if it is 1000 nm or less. The vaporization pressure gets lower as the pore throat gets tighter. In Hele–Shaw and microfluidic experiments, the recorded vapour pressures were relativity close to the phase-change pressures measured in bulk conditions. Due to their inner medium sizes, the existed capillary effects in the silicate glass models were not sufficient to result in shifted vapour or condensation pressures since they were larger than 100 nm. Additionally, the visualized phase change of propane in silica-glass models took place at pressures approximately equal to those computed by the Kelvin equation, based on their medium sizes and temperatures ($\approx 20\,^{ \circ } {\text{C}}$). Because of the presence of micro ($< 2$ nm) and meso (2–50 nm), the vapour pressures in the rocks were noticed to be lower than those measured in bulk cases. Figure 12 shows the measured condensation pressure in Hele–Shaw and microfluidic experiments, including the computed saturation pressures from the Kelvin equation. Figure 13 presents the vapour pressures that were measured in Hele–Shaw cells, microfluidic chips, and rock samples. Also, it compares the outcomes with the calculated phase-change pressures from the Kelvin equation. The average pore sizes of rocks were the median pore sizes, obtained from the pore size distribution analysis. On average, the vapour pressures in the rocks were 7% less than the bulk and calculated vapour pressures of propane.

**Figure 14:**
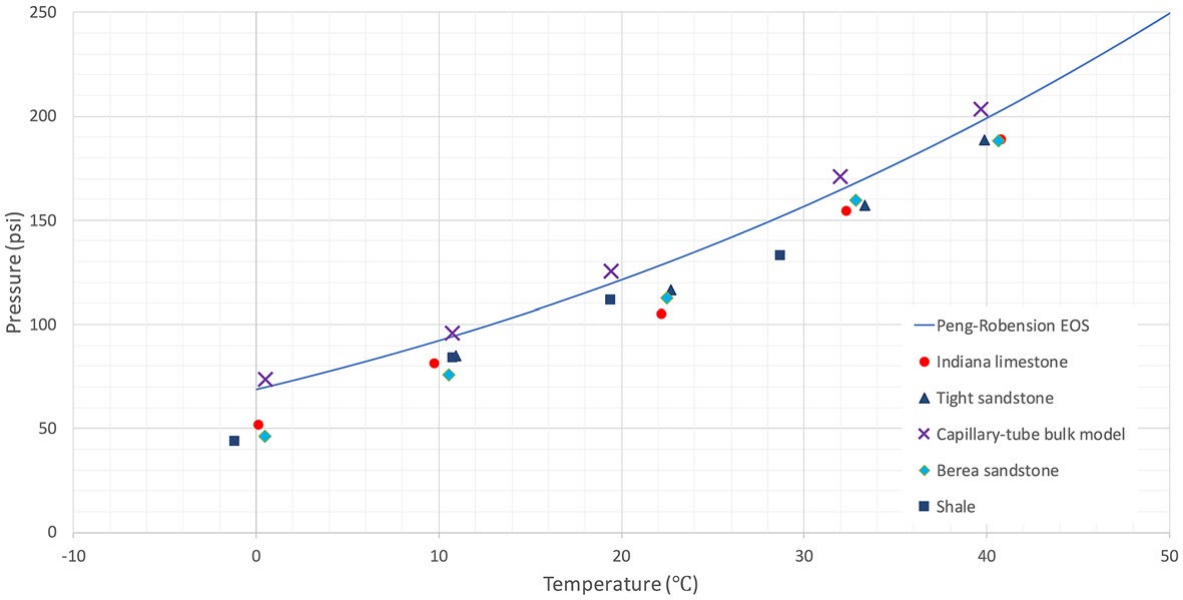
Measurement of vapour pressure in Berea sandstone, Indiana limestone, tight sandstone, and shale at various system temperatures. Each point represents the average of 3 experiments (3 trials with 3 core samples from identical reservoir rock blocks). Each point for the bulk condition represents the average of 3 trials.

The results were also compared with Peng–Robinson EOS which is one of the well-known pressure–volume–temperature (PVT) models in reservoir simulation that is used to predict the phase-change behavior of hydrocarbons. In our case, a phase envelope of the single-component hydrocarbon was generated by PR-EOS and compared with the measured phase-change pressures in the rock samples at various temperatures, ranging from 0 °C (273.15 K) to 40 °C (313.15 K). Since PR-EOS does not consider the capillary effect on its vapour-liquid equilibrium (VLE) calculation, the computed vapour pressures by the cubic EOS were identical to the bulk values and different from those measured in the reservoir rocks. Figure 14 illustrates a comparison between computed vapour pressures from PR-EOS and measured vapour pressures of propane in the bulk condition and reservoir rocks. Shifted vapour pressures were detected in the rocks at different temperatures, owing to the presence of nanopores ($\le$ 1000 nm). The reduction of phase-alteration pressures was estimated to be nearly 15% lower than those measured in bulk conditions and calculated phase-alteration pressures from PR-EOS.

## Conclusions and remarks

Surface characteristics of rock porous media, including capillary size, surface tension, and curvature of vapour-liquid interface, play an important role in controlling the thermodynamics and phase-alteration behaviour of liquids and gases. In order to achieve an accurate modelling of hybrid (with thermal) applications and sole solvent injection processes for oil recovery (and solvent retrieval), it is critical to understand the thermodynamics of the injected fluids (solvents) and originally existed fluids (heavy-oil, oil, condensate) in capillary medium conditions. The main objective of this paper was to compare our experimental observations with calculated vapour pressures from the Kelvin equation and Peng–Robinson EOS. Phase-change pressures of propane were investigated in capillary/porous media using Hele–Shaw glass cells, microfluidic silica-glass chips, and reservoir rock samples. The vapour pressures, measured in bulk conditions, were considered as benchmarks.

The propane vapour pressures measured with Hele–Shaw cells and microfluidic chips were comparable with those measured at bulk conditions. Additionally, they were identical with the computed saturation pressures from the Kelvin equation (Fig. 13). However, propane vaporized in rock samples at pressures lower than the bulk vapour pressure and computed values by 7%. Studying the phase-change pressure of propane at different temperatures allowed us to compare the experimental outcomes with one of sophisticated cubic equations of state (PR-EOS) in reservoir simulation. Due to the confinement effect, the measured vapour pressures in sandstone, limestone, tight sandstone, and shale were 15% (on average) less than those modelled by PR-EOS (Fig. 14) In reservoir scales, such shifted phase-change pressures could have an impact on the accuracy of reservoir fluid-dynamic simulations and history matching.

## Abbreviations

SAGD: Steam assisted gravity drainage
C5+: Pentane and higher carbon number hydrocarbons
EOR: Enhanced oil recovery
SOS-FR: Steam-over-solvents in fractured reservoir
CSS: Cyclic steam stimulation
EOS: Equation-of-state
SRP: Solvent retrieval process
PVT: Pressure–volume-temperature
CO_2_: Carbon dioxide
PR-EOS: Peng–Robinson equation-of-state
K: Kelvin
$v^{L}$: Liquid molar volume
$$r$$: Droplet radius
$$\gamma$$: Surface tension
$R$: Universal gas constant
$$T$$: Temperature
$P_{\infty }$: Vapour pressure at flat surface
$P_{r}$: Vapour pressure at curved interface
$T_{r}$: Temperature at porous medium
$T_{\infty }$: Temperature at bulk medium
$\Delta H_{vap}$: Heat of vaporization
$$V_{m}$$: Molar volume
$Z_{C}$: Critical compressibility factor
